# The State of Weight in Cystic Fibrosis: Understanding Nutritional Status and Individualizing Nutritional Care in the Modulator Era

**DOI:** 10.3390/nu17152533

**Published:** 2025-07-31

**Authors:** Sapna Khemka, Stacie Hunter, Jessica Jones, Keishla Valentín-Martínez, Christina B. Chadwick, Rosara Bass

**Affiliations:** 1 Division of Gastroenterology, Hepatology & Nutrition, Department of Pediatrics, Nationwide Children’s Hospital, The Ohio State University, Columbus, OH 43205, USA; 2 Children’s Health Children’s Medical Center, Dallas, TX 75235, USA; 3 Department of Pediatrics, University of Texas Southwestern, Dallas, TX 75390, USA; 4 Division of Gastroenterology, Department of Pediatrics, College of Medicine, University of Florida, Gainesville, FL 32610, USA

**Keywords:** cystic fibrosis, HEMT, nutrition, pediatrics

## Abstract

There is a well-established association between cystic fibrosis (CF) and malnutrition. Several comorbid conditions have also been associated with undernutrition in people with cystic fibrosis (PwCF). Highly effective modulator therapy has allowed for a paradigm shift altering disease progression and management. Modulator use has even been associated with acceleration of weight trajectory causing overnutrition, which can lead to cardiovascular and metabolic comorbid conditions. This review explores how nutritional status is evolving in the era of cystic fibrosis transmembrane conductance regulator (CFTR) modulators in people with CF, specifically in children. By synthesizing current research, we aim to support pediatric healthcare providers and nutritionists in delivering tailored, proactive nutritional care in this new therapeutic landscape.

## 1. Introduction

Nutritional status is associated with pulmonary outcomes and mortality in cystic fibrosis (CF), and thus, early identification and appropriate treatment of malnutrition has been a longstanding hallmark of CF care [1]. While cystic fibrosis transmembrane conductance regulator (CFTR)-related exocrine pancreatic insufficiency is a primary driver of malnutrition in CF, multiple other factors, both directly and indirectly related to CFTR dysfunction, contribute to growth and nutritional status in this population [2] Highly effective CFTR modulator therapies (HEMT) are associated with robust weight gain in many people with cystic fibrosis (PwCF), and the mechanism of modulator-associated weight gain is likely multifactorial and variable at the individual patient level. With HEMT-associated weight gain, as well as overall improved health and longevity in PwCF, overweight and obesity are also emerging problems [3]. In the modern era of CF, the term malnutrition may refer to undernutrition and overnutrition, both of which my negatively impact health outcomes in this population. Understanding specific factors which drive development of undernutrition and overnutrition in PwCF and recognizing that different factors may predominate in each individual will be essential for promotion of optimal overall health, wellness, and longevity in this population [1,4]. Presented here is a narrative review of the recent literature and landmark studies describing the state of nutritional status in the modern era of cystic fibrosis. We specifically acknowledge challenges related to both under- and overnutrition, thus highlighting the need for personalized nutrition therapy in people with cystic fibrosis.

## 2. Undernutrition in Cystic Fibrosis

Undernutrition is one of the earliest and best described manifestations of CF, and prevention of undernutrition is of paramount importance in the treatment of this disease [1]. Nutritional status, as measured by body mass index (BMI), has been found to be associated with improved pulmonary outcomes and overall survival in PwCF, and this relationship begins early in life [1,4,5,6,7]. Psoter et al. examined growth trajectories of children with CF during the first five years of life and found that the cohort of children with weight-for-length or BMI persistently >50th percentile had the highest mean forced expiratory volume in one minute percent of predicted (FEV_1_pp) at age six, while those with weight-for-length or BMI persistently below the 50th percentile had the lowest mean FEV_1_pp at age six [8,9]. CF nutrition guidelines, published in 2008, recommend that children with CF maintain a weight-for-length (age < 2 years) or BMI (age ≥ 2 years) greater than the 50th percentile for age and that adults with CF maintain a BMI of >22 or 23 kg/m^2^ for adult females and males, respectively [5]. These same cut points have been recommended by the 2024 European Guidelines on Nutrition Care for CF [10]. The concept of an unrestricted high-calorie, high-fat diet has been referred to as the legacy CF diet [11].

A primary and well-established driver of undernutrition in CF is exocrine pancreatic insufficiency (EPI), impacting approximately 85% of PwCF, and it is typically associated with severity of CFTR genetic mutation [12]. While pancreatic enzyme replacement therapy (PERT) can significantly improve nutrient absorption and nutritional status in PwCF, a number of additional factors impacting nutritional status in PwCF persist, including decreased small bowel pH, abnormal bile acid circulation, increased intestinal inflammation, and a pro-inflammatory colonic microbiome [13]. Individuals with CF and EPI have a resting energy expenditure 110–120% of predicted for age and sex and, thus, higher caloric needs to achieve age-appropriate growth in children or weight maintenance in adults [6]. CF-related diabetes (CFRD) and CF-related liver disease (CFLD) may also increase energy losses and increase metabolic demand. Despite a prescribed high-calorie diet, GI symptoms, which are highly prevalent among PwCF, may limit adequate intake. Additionally, intake may be limited in the setting of disordered eating behaviors, which may stem from significant pressure from family members or medical care surrounding weight and dietary intake [14].

While nutritional status in CF has historically focused on weight and BMI, length is another important growth metric in CF. Linear growth delay and/or attainment of a lower-than-predicted mid-parental adult height are common in PwCF, and stunting is associated with poorer lung function and independent risk of mortality [15,16]. Linear growth deficits are often seen in infancy and early childhood, prior to the onset of clinically significant pulmonary disease, and they persist despite early-life nutrition interventions [17]. Previous studies have shown that those with linear growth failure in early childhood, less than 10th% height for age (HFA) between 2 and 4 years of age, were at increased risk of also being <10th% height in adulthood [18].

## 3. Co-Morbid Conditions Associated with Undernutrition in PwCF

While multiple intrinsic factors related to CFTR dysfunction may be primary drivers of undernutrition in CF, PwCF are not immune from development of other comorbid conditions. Additionally, the presence of CFTR gene variants may impact the expression of other diseases. CF providers should remain vigilant when a patient’s growth fails to improve despite adequate nutritional optimization and enzyme replacement treatment. Failure to consider comorbid conditions can lead to delays in diagnosis and treatment.

### 3.1. Celiac Disease and Non-Celiac Gluten Sensitivity

Celiac disease (CD), an inherited autoimmune disorder resulting in enterocyte damage in the presence of gluten, can lead to chronic malabsorption and resultant growth failure. Celiac disease exhibits a prevalence 2–3 times higher in individuals with CF compared to the general population [19]. Some studies suggest that CFTR dysfunction predisposes individuals to CD by increasing the antigen load [20]. Nevertheless, coexistence of CF and CD is rare, and there is still no consensus for screening recommendations for CD. Interestingly, although a gluten-free diet is the standard approach for treatment, ivacaftor has been suggested as a potential therapeutic option. Studies have shown that ivacaftor reduces inflammation and induces gluten tolerance by attenuating gliadin’s negative impact on CFTR function [21]. However, there are also individuals who have been identified with celiac disease while taking ivacaftor [22].

Celiac disease may impact weight through impaired nutrient absorption due to villous blunting. Weight may also be impacted in non-celiac gluten sensitivity, even in the absence of intestinal mucosal changes. While individuals with NCGS do not exhibit intestinal damage to wheat proteins, they experience intestinal and extra-intestinal symptoms related to gluten ingestion, which may result in weight loss due to inadequate oral intake due to GI symptoms or avoidance of trigger foods [23].

### 3.2. Eosinophilic Esophagitis

Eosinophilic esophagitis (EoE) is an allergic/immune-mediated condition in which eosinophilic inflammation in response to a food antigen inappropriately develops in the esophagus, leading to dysfunction and fibrosis, and it has been reported in PwCF [24]. A retrospective cross-sectional study of children with and without EoE found that the rate of CF comorbidity was 0.9% in patients with EoE and 0.05% in patients without EoE [25]. Moreover, prevalence of EoE has been estimated to be approximately 6 times higher in PwCF [26]. Undernutrition is a common symptom of EoE and is typically due to inadequate intake secondary to dysphagia. Treatment of eosinophilic esophagitis is individualized for each patient, and therapeutic options include dietary allergen elimination, high-dose proton pump inhibitors, swallowed steroids, and biologic therapies [27]. Comorbid CF may impact therapeutic decision making for EoE for multiple reasons; for example, PwCF may already have a high oral and inhaled steroid burden, which may impact decision making regarding topical steroids. While chronic use of systemic steroids can result in suppression of growth in children, weight gain with centripetal redistribution of fat, muscle wasting, and osteoporosis, topical steroids are most often associated with the side effects of oral and/or esophageal candidiasis [28,29]. Additionally, there is risk associated with imposing significant dietary limitations in individuals who already have increased caloric needs, as exists in CF.

### 3.3. Inflammatory Bowel Disease

Crohn’s disease, indeterminate colitis, and ulcerative colitis, referred together as inflammatory bowel disease (IBD), are disorders characterized by chronic, immune-mediated intestinal inflammation. IBD has been described in PwCF, with reported higher prevalence (1.3%) than in the general population (0.8%) [30]. Interestingly, despite the higher prevalence of IBD in PwCF, exome sequencing has revealed a strong association between the CFTR delF508 variant and reduced IBD risk [31]. The discordance between prevalence and theoretical genetic risk highlights the need for mechanistic studies to better understand the factors driving development of IBD in PwCF. As with other comorbid gastrointestinal conditions, clinical symptoms of IBD can mimic common GI symptoms seen in PwCF, including abdominal pain, stool abnormalities, and weight loss, and the symptom overlap may delay IBD diagnosis, particularly in Crohn’s disease. Treatment of IBD typically involves immune mediation, which can impact infection risk. Additionally, IBD treatment can be augmented or, in some cases, treated by dietary interventions, which focus on nutrient-dense, minimally processed foods and have been shown to restore microbial balance, reduce systemic inflammation, and improve nutrient absorption in these patients [32]. As with other GI disorders, the risks and benefits of different treatment options must be weighed independently and in the context of other CF-specific therapies.

### 3.4. Cystic-Fibrosis-Related Diabetes (CFRD)

Cystic-fibrosis-related diabetes (CFRD) is a distinct form of diabetes seen in PwCF and is characterized by insulin insufficiency as the result of pancreatic islet dysfunction. CFRD prevalence increases with age, impacting over 50% of adults with CF. CFRD is independently associated with increased morbidity and mortality [33,34]. Current CF Foundation (CFF) guidelines recommend the use of insulin as the primary treatment of CFRD [35]. While insulin insufficiency impacts absorption of carbohydrates, recommendations for CFRD management have historically not recommended carbohydrate restriction due to risk of perpetuating underweight status [34]. Instead, carbohydrate counting, a method that quantifies carbohydrate intake, can be used alongside insulin therapy to help maintain stable blood sugar levels [34,35].

### 3.5. Advanced CF Liver Disease

Advanced CF liver disease (aCFLD) is defined as having one or more of the following: nodular liver, advanced fibrosis, multilobular cirrhosis with or without portal hypertension, or noncirrhotic portal hypertension [36]. Due to these underlying pathogenic changes, individuals with aCFLD have increased metabolic demands and increased inflammatory burden, further perpetuating risk of undernutrition. To address this, the CFF recommends that people with aCFLD receive nutritional assessment and liver-specific nutrient deficiencies every 6 months by a dietitian experienced in CF [37,38]. Focus should be placed on optimizing nutrition and monitoring for other co-morbidities that these individuals are at risk for that could lead to nutritional risk, including the comorbid conditions described above. High prevalence rates of fatty acid (FA) composition abnormalities have been reported in CF, thought to result from disrupted metabolism of essential fatty acids (EFAs) in the setting of liver disease [37,38]. While treatment of EFA deficiencies through targeted nutritional interventions are attempted, deficiencies often persist even with adequate therapy [39].

## 4. Overnutrition in Cystic Fibrosis

### 4.1. Overnutrition in CF Prior to Widespread HEMT Use

The prevalence of overweight and obesity in CF have dramatically increased over the past two decades, with upward trajectory predating widespread eligibility for HEMT. In the US CF Patient Registry, the percentage of adults with CF meeting criteria for overweight or obesity has more than doubled over the past 20 years (17.1% in 2003, 41.4% in 2023). Additionally, in the most recent three years of available registry data (2020–2023), the prevalence of overweight increased from 26.7% to 28.4 and obesity increased from 10.3% to 13.0% [40,41]. Analysis of the Italian CF patient registry from 2016–2021 found the prevalence of overweight increased from 10% to 17%, and the prevalence of obesity increased from 2% to 3.7% among adults with CF during that time period [3]. The prevalence of overweight in CF is comparable to the prevalence of obesity among U.S. children and adolescents of 20% [42].

In PwCF, overweight and obesity have traditionally been associated with factors consistent with more mild disease, including CFTR genotype associated with pancreatic sufficiency or mild CFTR dysfunction, higher FEV_1_pp, older age, and fewer pulmonary exacerbations [5,6]. Despite the higher prevalence of overweight and obesity in PwCF with a more mild disease phenotype, they are still seen in PwCF with more severe disease and EPI [3,6,7]. In a single-center, cross-sectional database analysis of adults with CF from 2015–2017, 25% of adults with severe CFTR genotypes (associated with pancreatic insufficiency and/or little to no functional protein) had BMI ≥ 25 kg/m^2^ [6,9].

### 4.2. Acceleration of Weight Trajectory with HEMT and Potential Mechanisms

**Figure 1:**
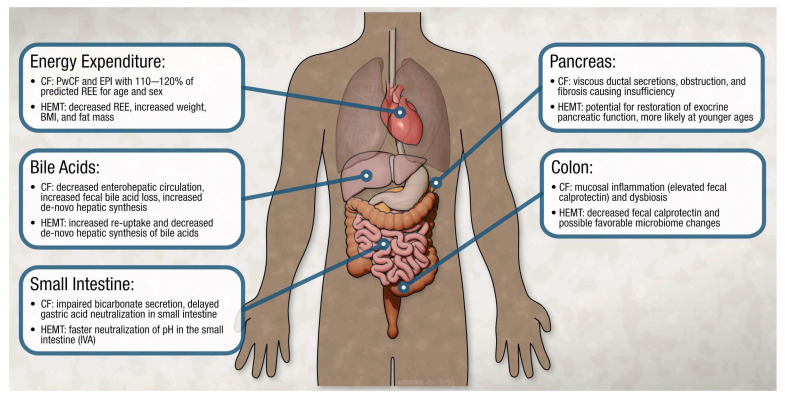
Effect of highly effective modulators on factors impacting nutritional status in people with cystic fibrosis. This figure demonstrates how cystic fibrosis (CF) affects the gastrointestinal tract at various levels, impacting nutritional status. The biochemical alterations to normal physiology in CF is targeted by highly effective modulator therapy (HEMT) at a molecular level as described. Abbreviations: resting energy expenditure (REE); people with cystic fibrosis (PwCF); exocrine pancreatic insufficiency (EPI); body mass index (BMI); ivacaftor (IVA).

While overweight and obesity were increasing in prevalence prior to widespread eligibility for HEMT, HEMT-associated weight gain has likely accelerated these changes. In a clinical effectiveness study of ivacaftor in adults that examined anthropometrics at baseline and 5.5 years after initiation of ivacaftor, the prevalence of overweight increased from 16% to 25% and obesity from 8% to 11% [10]. Real-world single-center data examining anthropometrics and body composition pre- and post-elexacaftor/tezacaftor/ivacaftor (ETI) identified that the prevalence of overweight increased from 19% to 29% and obesity increased from 3.6% to 5.5% [43]. The mechanism underlying HEMT-associated weight gain is likely multifactorial and variable between individuals. The following are specific potential mechanisms that have been explored in the literature (Figure 1).

### 4.3. Changes in Energy Expenditure

Energy expenditure exceeding caloric intake can contribute to growth failure and malnutrition, and such an imbalance may occur in CF due to insufficient dietary intake, fat malabsorption, and higher resting energy expenditure (REE) needs [44]. A study comparing children with CF to a control group (n for each group = 25 ages 6–9) found that the cohort with CF had resting energy expenditure 9% higher than controls that was unrelated to disease genotype of pulmonary function [44]. Moudiou et al. (2007) examined resting energy expenditure in youths and young adults with CF (ages 9–24 years, n = 38) and identified that energy needs were higher than expected REE for age and sex in this population, and energy needs were higher in participants with pancreatic insufficiency than sufficient (t = 2.352 in the two-tailed paired Student’s *t*-test), but there was no difference in energy expenditure when compared by differences in pulmonary function [6].

While specific differences in pulmonary function have not been shown to be associated with energy expenditure differences in CF, the overall inflammatory burden in CF contributes to the increased energy needs, particularly due to the chronic bacterial colonization and airway inflammation in the respiratory tract [45]. HEMT has been shown to reduce systemic inflammation, which could theoretically allow for conservation of energy [45]. Gur et al. (2022) (n = 18, median age 18.6 years) demonstrated an increase in bone mineral density and body mass index, evaluated by dual-energy X-ray absorptiometry (DEXA) scans, as well as increased exercise capacity, evaluated by spirometry, lung clearance index, sweat test, six minute walk test, and cardio-pulmonary exercise test, in participants with CF treated with ETI [46].

### 4.4. Changes in Exocrine Pancreatic Function

The exocrine pancreas is responsible for production and secretion of digestive pro-enzymes, fluid, and bicarbonate which, when secreted into the small intestine, facilitate nutrient digestion [47]. CFTR is expressed on ductal cells of the pancreas, which are specifically responsible for chloride-dependent fluid and bicarbonate secretion. Absent or insufficient CFTR results in viscous pancreatic secretions, resulting in ductal obstruction, inflammation, fibrosis, and acinar destruction [48]. This process begins in utero, often resulting in exocrine pancreatic insufficiency (EPI) at birth or early in life, with 85% of PwCF developing EPI by one year of age [12]. Classic symptoms of EPI include weight loss due to malabsorption of nutrients, flatulence, bloating, dyspepsia, and steatorrhea [12]. Steatorrhea occurs when lipase secretion is reduced by at least 90% [49]. PwCF and EPI require pancreatic enzyme replacement therapy (PERT) with all fat-containing meals or snacks to facilitate sufficient nutrient absorption, as well as monitoring and often additional replacement of fat-soluble vitamins [12].

Restoration of pancreatic function, indirectly measured by fecal elastase increasing from <200 µg/g to >200 µg/g, is not universal in PwCF treated with HEMT. However, several case studies and case series have demonstrated improvement and even restoration of pancreatic function in a subset of PwCF treated with ivacaftor [50,51]. Nichols et al. demonstrated that participants 12 to 24 months old increased fecal elastase and decreased immunoreactive trypsinogen at 24 weeks after starting treatment, indicating improvement in pancreatic function biomarkers. This supports ivacaftor’s ability to preserve exocrine pancreatic function when started early in life [52]. In participants who developed EPI above the age of 6 years, the mean increase in fecal elastase from baseline was 299 µg/g, and the mean time to pancreatic sufficiency was 5 years [51]. More recent reports of restoration of pancreatic function have also been seen with ETI. In a prospective observational study by Stastna et al. of adults with CF examined before and after initiation of ETI (n = 29, median age 29.1), 82.8% of participants met criteria for EPI at baseline based on a fecal elastase level of <200 µg/g. After 24 months of treatment with ETI, participants had statistically significant improved nutritional parameters, including an increase in BMI by 1.20 kg/m^2^, albumin by 2.81 g/L, and prealbumin by 0.06 g/L, as well as a decrease in total protein by 3.29 g/L. Participants also reported decreased bowel movements, resulting in self-modifications of daily PERT dosing in response to their change in stool pattern. Only one patient (4.5%) developed pancreatic sufficiency with an increase in fecal elastase of >200 µg/g [53].

### 4.5. Changes in Intestinal Absorption

Neutralization of gastric acid is essential for nutrient digestion and absorption in the small intestine and is achieved by secretion of pancreatic bicarbonate into the duodenum [2]. Adequate activation of lipase requires an intestinal pH of greater than 4. Additionally, enteric coating of PERT is designed to dissolve at a pH of 5 [49,54]. Pancreatic phospholipase A2 requires a pH of 7–9 for activation and leads to lyso-lipid partitioning, allowing for fat hydrolysis, specifically triglycerides [55,56]. Impaired pancreatic bicarbonate secretion in individuals with CF and EPI leads to delayed buffering of gastric acid and more distal achievement of neutral pH compared to individuals without CF [49,54].

Gelfond et al. examined pH and transit throughout the intestine in 10 adults (median age 35.7) with CF before and one month after initiation of ivacaftor [57]. The mean time to reach and sustain pH > 5.5 in the small intestine after gastric emptying was 40 min pre-ivacaftor and 8 min post-ivacaftor [57].

Small-intestinal bacterial overgrowth (SIBO) has also been implicated in up to 40% of PwCF, attributed to impaired motility and viscous mucosa, both serving as an anchor for bacteria and impairing intestinal Paneth cell defenses against bacterial overgrowth [58]. If untreated, SIBO may result in nutrient malabsorption, including fats, fat-soluble vitamins, and vitamin B12. Folate may even be increased due to bacterial synthesis of folic acid [58].

### 4.6. Changes in Bile Acid Metabolism

In PwCF, bile acid reabsorption is impaired due to the dehydrated and viscous mucous layer present on the intestinal luminal surface, resulting in increased fecal bile acid excretion and decreased enterohepatic circulation compared to a control population [59]. Diarrhea is a resulting symptom due to high colonic bile acid concentrations, stimulating excess secretion of water and electrolytes as well as contractions [60]. Without reabsorption of bile acids, the nuclear farnesoid X receptor (FXR) pathway is impaired, leading to decreased release of fibroblast growth factor 19 (FGF19), a factor that triggers signaling pathways to suppress bile acid synthesis. The impairment leads to upregulation of bile acid synthesis, resulting in biliary cirrhosis [59]. Colonic dysbiosis is also linked to bile acid dysmetabolism in the gut, creating lipophilic bile acid metabolites that bind to and activate nuclear receptors, including activation of the FXR pathway, increasing FGF19, and decreasing bile acid synthesis [61]. The metabolites, however, are recycled into the enterohepatic circulation to maintain a functional bile acid pool [61].

Furthermore, PwCF develop choline deficiency due to loss of bile phosphatidylcholine in the feces in the setting of EPI. Pancreatic phospholipases are required to cleave phosphatidylcholine into lyso-phyosphatidylcholine, allowing for re-uptake of choline [62]. Impaired pancreatic bicarbonate and phospholipase secretion results in increased fecal phosphatidylcholine loss and defective enterohepatic cycling, ultimately causing low plasma choline. Lung tissue upregulates choline accumulation via high-density lipoproteins [62].

Schnell et al. conducted a prospective observational study (n = 20, mean age 20) of bile acid profiles. PwCF had significantly higher total bilirubin, conjugates, and metabolites compared to control participants. There was no significant change in total serum bile acid concentration in PwCF before and after 6 months of ETI [63]. 

### 4.7. Changes in Colonic Inflammation and Microbiome

Defective CFTR in colonic epithelial cells causes impaired bicarbonate secretion, eliminating a key component in mucous solubilization [64]. As a result, intestinal mucous remains dehydrated and viscous, which predisposes to dysbiosis and mucosal inflammation. An association between antibiotic use and gut dysbiosis exists due to reduced microbiome diversity and richness [65]. This is similar to the pathophysiology noted in the pulmonary system resulting in frequent opportunistic pulmonary infections in patients with CF [66]. Bruzzese et al. examined fecal calprotectin and nitric oxide (NO) concentrations, both non-invasive markers of colonic inflammation, in children with CF (n = 75, mean age 9.3) and found that calprotectin and NO concentrations were higher in PwCF compared to controls [67]. Calprotectin and NO concentrations were lower one month after oral probiotic administration of Lactobacillus rhamnosus GG compared to baseline (calprotectin 210 vs. 140, *p* < 0.01; nitric oxide 21.2 vs. 4.3, *p* < 0.05) [67].

Hevilla et al. completed a prospective observational study in which fecal calprotectin and fecal nitrogen concentrations were examined as a marker of intestinal inflammation in a cohort of adults with CF (n = 34, mean age 39.7) prior to and after one year of treatment with ETI. Lower concentrations of fecal calprotectin (623.1 vs. 96.9, *p* = 0.048) and fecal nitrogen (7.2 vs. 3.7, *p* = 0.021) were identified at follow up, suggesting a favorable change in colonic inflammation and microbiota modifications one year after treatment with ETI [68].

### 4.8. HEMT and Body Composition

When considering potential effects of HEMT-associated weight gain, it is important to consider body composition, or the distribution of weight between fat mass and fat free mass. In CF, the relationship between BMI and pulmonary function has largely been shown to be driven by differences in lean body mass, or fat-free mass (FFM), as opposed to fat mass (FM) [43,69,70]. In fact, excess adiposity in the form of normal-weight obesity, has been found to be inversely associated with lung function in CF PwCF [71].

The impact of HEMT on body composition has been examined longitudinally with mixed results. Two studies examining body composition changes with ivacaftor both found increases in FM associated with treatment, but only one identified changes in FFM [72,73]. A single-center study of body composition before and after 12 months of ETI, found increases in both FM and FFM, but increases in FM were greater than those in FFM [74]. In a single-center, double-blind placebo-controlled crossover study of ivacaftor in adults, changes in FFM were seen during the first 28 days of treatment, followed by changes in BMI and FM without changes in FFM in the following 5 months [75]. In a study of body composition of 62 youths treated with ETI, absolute FM and FFM were higher at 6 months of ETI as compared to pre-ETI baseline. Notably, no further changes in FM nor FFM were seen in 32 participants who continued the study for an additional 6 months [76]. In a retrospective study analyzing body composition on chest CT scans, ETI was associated with increases in adipose tissue ratios but not with muscle ratios, and these effects were most prominent in PwCF who were underweight pre-treatment [77].

## 5. Impact of Overnutrition in PwCF

### 5.1. Overweight and Obesity and Pulmonary Function

From the 2023 CFF Patient Registry Data Report, pulmonary function has been positively associated with increase in BMI until a certain threshold of about 26 kg/m^2^ [33]. As BMI continues to increase, FEV_1_pp does not continue to improve but rather reaches a plateau. The lack of a continued relationship between increasing BMI and pulmonary function suggests that overweight and obesity status have no additional benefits on pulmonary function as compared to normal weight [33].

### 5.2. Cardiovascular and Metabolic Disease in CF

With increasing longevity, as well as increasing prevalence of overweight and obesity in PwCF, the impact of traditional cardiometabolic risk factors in the general population are considered in this population. Frost et al. found a higher prevalence of major adverse cardiac events in PwCF than a matched non-CF cohort [78]. Among the cohort with CF, older age and presence of hypertension, hypercholesterolemia, and diabetes were associated with major cardiac events [78]. Lipid levels are often low in PwCF, even after HEMT, but this may be falsely reassuring, as major cardiac events have been reported in PwCF with normal LDL levels [79,80]. Changes in lipid levels have been seen in some studies after use of HEMT [81,82]. It is recommended that PwCF undergo cholesterol and lipid monitoring as per age-appropriate practices for the general population [83].

### 5.3. CF Hepatobiliary Involvement

Hepatic steatosis is the most common hepatic manifestation in PwCF and is considered a feature of CF-related hepatobiliary involvement (CFHBI). Some studies indicate that although hepatic steatosis is prevalent in CF, it may develop independently of typical malnutrition indicators such as BMI and fat stores. Interestingly, patients with hepatic steatosis tend to show greater muscle mass reserves, which might suggest that the presence of fatty liver in CF may not always correlate with the conventional signs of malnutrition [84]. This highlights the complexity of liver disease in CF and indicates that malnutrition may not be the sole factor contributing to hepatic steatosis in these patients. Bernhard et al. demonstrated that oral choline supplementation for almost 3 months decreased hepatic steatosis from 1.58% to 0.84% (*p* < 0.01) in 10 adult males with CF [85]. With overall increasing rates of overweight status and obesity in the CF population, hepatic steatosis likely also is and will continue to be seen in the setting of developing metabolic-dysfunction-associated steatotic liver disease, or MASLD, in this population, like in the general population [33].

### 5.4. Type 2 Diabetes

In contrast to CFRD, the pathophysiology of Type 2 Diabetes (T2D) is driven by insulin resistance, not insulin secretion. In the general population, Type 2 Diabetes generally presents insidiously in adulthood, with overnutrition and increased abdominal adiposity as risk factors for development of T2D. Additionally, a family history T2D is a strong risk factor for T2D development [86]. While CFRD and T2D are unique entities, a family history of T2D is also associated with increased risk of CFRD [87]. With increasing prevalence of overnutrition and abdominal adiposity in PwCF, it is likely that these may further compound risk of diabetes development, particularly in those who also have a family history of T2DM.

## 6. Evolving Practices in Nutritional Care for PwCF

While dietary counseling in CF has historically prioritized fat and calorie intake to promote optimal health, with improved longevity and pulmonary health, as well as increased prevalence of overweight and obesity, dietary guidance in CF now focuses on intake of nutrient-dense foods and energy intake, as appropriate, to support normal, age-appropriate growth as opposed to specific caloric targets [88]. It is also important to recognize that the CF “legacy diet” also promotes eating behaviors to promote weight gain, which may become maladaptive and obesogenic when energy needs change (i.e., after initiation of HEMT). Thus, approaches to nutrition counseling in CF must also focus on eating behaviors, including mindfulness or intuitive eating. Care teams should also consider social factors including family dynamics, socioeconomic status, and food insecurity when creating individualized nutrition plans for PwCF [88].

### 6.1. Weight Loss Medications in PwCF

Glucagon-like peptide-1 (GLP-1) agonists, which have been used for the general population for improved weight loss and glucose control, have recently been used in adults with CF, as documented in multiple case series [89,90]. Based on the reported uses of GLP-1 agonists so far, these medications have not only been associated with improved weight loss and glucose control but also improved lung function in adults with CF. It was found that there was a 7% to 19% weight reduction in four out of five individuals over a 2-year treatment regimen. Traditionally, this type of medication had been used sparingly in the management of CF-related diabetes [90].

### 6.2. Eating Disorders/ARFID

Studies have shown that young people with diet-related chronic health conditions are at increased risk for developing disordered eating behaviors [91]. PwCF often face unique challenges related to nutrition, growth, and body image. Intense focus on food, weight, and shape as part of a care plan and rapid changes in weight related to new therapies (insulin, PERT, HEMT) can create a perfect storm for the development of disordered eating patterns. This highlights the importance of a multidisciplinary team, including psychologists and dietitians, in managing PwCF. Some studies suggest that appetite stimulants such as megestrol acetate, cyproheptadine, dronabinol, and mirtazapine may be helpful for PwCF and anorexia nervosa [92].

## 7. Conclusions

Nutritional status continues to be of paramount importance in the treatment of PwCF. While trends toward higher weight and BMI have been seen among PwCF over the past decade, accelerated by use of HEMT, a subset of PwCF remain underweight. With risks associated with both under- and overnutrition, an individualized care plan is warranted both in the consideration of etiologies and treatment to optimize an individual’s nutritional status. Additionally, while nutrition has been primarily viewed as a tool for optimizing pulmonary health, as PwCF live longer and healthier lives, it will be essential to balance the potential benefits of weight on pulmonary function with emerging risks of obesity-related co-morbidities.
